# Online Meta-Recommendation of CUSUM Hyperparameters for Enhanced Drift Detection

**DOI:** 10.3390/s25092787

**Published:** 2025-04-28

**Authors:** Jessica Fernandes Lopes, Sylvio Barbon Junior, Leonimer Flávio de Melo

**Affiliations:** 1 Department of Electrical Engineering, Londrina State University (UEL), Londrina 86057-970, Brazil; leonimer@uel.br; 2 Department of Engineering and Architecture, Università degli Studi di Trieste (UNITS), 34127 Trieste, Italy; sylvio.barbonjunior@units.it

**Keywords:** change-point detection, CUSUM, meta-learning

## Abstract

With the increasing demand for time-series analysis, driven by the proliferation of IoT devices and real-time data-driven systems, detecting change points in time series has become critical for accurate short-term prediction. The variability in patterns necessitates frequent analysis to sustain high performance by acquiring the hyperparameter. The Cumulative Sum (CUSUM) method, based on calculating the cumulative values within a time series, is commonly used for change detection due to its early detection of small drifts, simplicity, low computational cost, and robustness to noise. However, its effectiveness heavily depends on the hyperparameter configuration, as a single setup may not be universally suitable across the entire time series. Consequently, fine-tuning is often required to achieve optimal results, yet this selection process is traditionally performed through trial and error or prior expert knowledge, which introduces subjectivity and inefficiency. To address this challenge, several strategies have been proposed to facilitate hyperparameter optimizations, as traditional methods are impractical. Meta-learning-based techniques present viable alternatives for periodic hyperparameter optimization, enabling the selection of configurations that adapt to dynamic scenarios. This work introduces a meta-modeling scheme designed to automate the recommendation of hyperparameters for the CUSUM algorithm. Benchmark datasets from the literature were used to evaluate the proposed framework. The results indicate that this framework preserves high accuracy while significantly reducing time requirements compared to Grid Search and Genetic Algorithm optimization.

## 1. Introduction

The growing technological evolution in several domains has resulted in a large amount of data production over time. Massive amounts of data have been generated from various applications, such as application logs, sensor devices, and metrics monitoring, among others. In most domains, time-series analysis proves to be a needed and valuable strategy to provide early change detection, which provides adequate decision-making. For instance, advanced sensing technologies and methodologies, such as those proposed by [1], have highlighted the importance of high-precision change-point detection in diverse applications, including industrial monitoring and healthcare systems [2]. However, each time-series scenario demands methods capable of modeling an accurate analysis and adapting to each changing behavior. Meanwhile, traditional methods often face challenges in adapting to the dynamic nature of real-world scenarios, necessitating accurate strategies.

In offline data analysis, it is generally assumed that the data are generated by a nonstationary distribution, which means the behavior has not been the same throughout the data stream [3]. In this way, a notable problem that has been introduced by time-series studies is that changes might occur over time, which can lead to poor prediction performance and ineffective decisions if the set of hyperparameters is not adequate. However, due to the fundamental challenges in time-series patterns, in a real-world scenario, time series show highly nonlinear and nonstationary patterns [4].

Abrupt structural changes occur in many fields. To detect them, statistical analysis aims to identify these time points [5]. In general, the change-point analysis detects whether or not the change has occurred [6]. Due to structural changes that time series suffer from, the quantification and detection of these change points are considered a challenge. Considering change-point analysis and traditional techniques, the Cumulative Sum (CUSUM) algorithm has been widely used for detecting mean shifts since first introduced [7]. Due to its simplicity and ease of use beyond efficiency, it has been considered to be a base for several other proposed approaches, as it is robust to noise and has a low computational cost [8,9,10,11].

CUSUM is designed to identify the discrepancy among sequential numerical data. However, its performance can decrease due to missing representative data or incorrect hyperparameter configuration [12]. Thus, achieving good performance usually requires fine-tuning, which involves expert knowledge [13]. In this sense, to choose hyperparameters, it is necessary to solve fundamental problems in different scenarios to input the correct set, which is both time-consuming and abstract. To avoid a trial-and-error approach for hyperparameter configuration, different cases involving data analysis have been successfully solved with Automated ML (AutoML) over the years [14,15,16,17,18].

AutoML has been widely adopted in machine learning to enhance performance metrics [19], specifically in hyperparameter optimization (HPO), to tackle the inherent complexity and computational intensity of tuning machine learning models [18,20]. In the context of time series, AutoML methods have gained significant acceptance in forecasting [21] and data cleaning [22,23]. Time-series data originate from diverse sources, exhibiting significant variability, while user objectives for drift detection also differ. No single detection method consistently performs well across all scenarios, and tuning algorithm parameters is often complex and unintuitive without expert guidance. Moreover, the high cost of obtaining ground truth data makes the evaluation of detection algorithms a challenging task [24,25].

To do so, in this paper, we aim at using an AutoML-based solution to recommend a set of hyperparameters to CUSUM focusing on high predictive performance and low computational time consumption. In this manner, we propose a framework built on meta-learning (MtL) to automatically configure hyperparameters without human intervention. This strategy aims to make decisions in an objective, data-driven, and automated way [26]. Oriented by metadata information, a suitable set of hyperparameters may be automatically chosen based on performance in a certain scenario. The automatic process reduces human efforts and improves performance, adapting to the problem at hand [27]. In this context, MtL has been widely used as a strategy in various optimization problems [28,29,30].

Recent studies have applied MtL strategies in the context of change-point detection, leveraging their ability to adapt dynamically to evolving data streams. For instance, MtL techniques have been successfully employed for HPO in drift detection methods, significantly reducing manual interventions and computational costs associated with traditional trial-and-error strategies. Silva et al. [20], for example, utilized a lightweight unsupervised MtL strategy specifically designed to enhance drift detector tuning, demonstrating improved adaptability and efficiency in streaming data environments. Martins et al. [25] also applied meta-learning to dynamically adjust active learning parameters in data stream classification, illustrating substantial performance improvements under changing data distributions. However, despite these advancements, existing MtL applications primarily focus on generalized drift detection or classifier performance tuning. In contrast, our proposed approach uniquely integrates a dedicated meta-learning mechanism specifically tailored to automate hyperparameter recommendation for the CUSUM algorithm, directly addressing the gap of real-time parameter adjustment in statistical change-point detection tasks. Thus, this work presents a novel meta-recommendation framework explicitly designed to optimize CUSUM parameters, enabling more responsive, accurate, and computationally efficient drift detection in diverse real-world scenarios.

Thus, given the dependence of the CUSUM algorithm on hyperparameters, we suggest an MtL framework to dynamically recommend a suitable set of hyperparameters to CUSUM for change-point detection in time series. Our framework benefits from the collected signal based on a sliding window to determine the most suitable configuration by extracting relevant meta-features that characterize the underlying time-series dynamics. These meta-features are used to build a meta-learning model that maps time-series characteristics to a set of CUSUM hyperparameters. Our pipeline significantly reduces the time consumption in comparison to the other tested methods in order to find out the suitable hyperparameters, hence, enabling the use of the CUSUM in online environments with complex time-series behavior.

Here, we evaluated the two important aspects (predictive performance and time consumption) from the hyperparameters’ recommendation by MtL to the CUSUM prediction. To have a fair comparison, we analyzed the performance of the proposed framework in comparison to the Grid Search (GS) and Genetic Algorithm (GA) optimization approaches. The CUSUM’s hyperparameters were varied to analyze their relationship with the two main performance metrics. We summarize the main contributions of this work as follows:

1. Proposed an MtL framework to provide a guided selection of a suitable set of hyperparameters for CUSUM, which resulted to be a significant improvement in drift detection performance.
2. Analyzed the two-way relationship of drift detection performance and time consumption while varying methods and algorithms (ML baseline).
3. With the proposed automatic fine-tuning, the traditional CUSUM can be applied in scenarios with stationary time-series changes and even in online scenarios.

We believe that our contribution has potential for real-world applications where timely and accurate detection of change points is critical. In particular, industrial and engineering systems increasingly rely on data-driven monitoring for anomaly detection and predictive maintenance. For example, Sun et al. [11] applied CUSUM-based strategies for fault detection in engines, showcasing the algorithm’s effectiveness in high-reliability systems. Similarly, Kravchik and Shabtai [13] employed change detection methods for identifying cyber-attacks in industrial control systems, where early drift detection is vital for preventing disruptions. The proposed meta-learning approach, similar to Martins et al. [25], enhances these applications by eliminating the need for manual or heuristic hyperparameter selection, which is often infeasible in online or resource-constrained environments. By significantly reducing the time required to identify optimal configurations, our framework supports real-time responsiveness and adaptability, making it suitable for deployment in Industry 4.0 scenarios, such as smart manufacturing, energy systems, and autonomous infrastructure monitoring.

The remainder of this work is organized as follows: Section 2 gives an overview of mean shift detection, focusing on hyperparameters’ optimization, CUSUM, and MtL. Section 3 defines the task and its configuration steps, while Section 4 presents our proposed framework to recommend a proper set of hyperparameters for the CUSUM algorithm, the material used for experiments, the techniques, and metrics adopted. Section 5 shows the results and raises a discussion about them and their advantages and drawbacks. Section 6 presents the main conclusions and directions for future work.

## 2. Related Work

Time-series analysis has been widely explored in several fields. With respect to the mean shift task, it might occur in several learning problems. Thus, techniques have been developed to identify parts of the distribution in which the relationship between features and targets changes, which is known as concept drift detection [31]. Regarding the univariate distribution, the traditional techniques in detecting quickly abrupt changes have been well studied (e.g., CUSUM) [11,32]. In general, these methods are built as signal analyzers, which are based on the classifier’s performance or incoming data. However, the distribution changes over time, requiring updates or even retraining of models [33]. As mentioned above, the presence of concept drift may cause a significant drop in the accuracy of the models. The presence of changing distribution boosted researchers to develop flexible strategies and an adaptive mechanism to tackle several time-series scenarios. Taking into account the fine-tuning methods, MtL has been applied as an intelligent solution to face these challenges [1,34,35,36].

Designing and tuning ML algorithms requires substantial expertise. Hyperparameter optimization methods, such as Grid Search, Random Search, and Bayesian Search, are commonly employed to improve performance metrics [37]. Tuning involves selecting the hyperparameters for an ML model to achieve suitable performance on a specific task. In contrast, fine-tuning refers to the process of taking a pre-trained model and making minor adjustments to its parameters to adapt it to a new but related task. This approach leverages the existing knowledge of the model, allowing efficient adaptation with typically less data and computational resources [38]. Considering the plethora of possible configurations, automatic recommendation strategies often reduce processing time by pruning the search space, aiming to quickly identify the set of hyperparameters. When building ML models for a specific task, we might take advantage of the experience of related tasks, which can assist the decision-making process. MtL is concerned with observing and learning from ML experiments to provide better predictive performance. For that, it correlates the problem space and the algorithm space with the goal of maximizing performance.

Recent research has focused on improving hyperparameter optimization (HPO) for time-series methods. Jati et al. [21] proposed H-Pro, a technique that leverages data hierarchies to address test-validation mismatches in temporal cross-validation. Zhang et al. [39] introduced SSL-HPT, a self-supervised learning framework that significantly accelerates HPO compared to search-based methods. Wu et al. [40] developed an automated HPO approach using parallel genetic algorithms, demonstrating improved performance and reduced time costs for IoT time-series prediction. Fristiana et al. [41] conducted a comprehensive survey of HPO methods for deep learning in time series classification, finding that metaheuristic algorithms and Bayesian optimization are commonly used. These studies highlight the importance of efficient HPO techniques in enhancing the performance of time-series models across various domains, including forecasting, classification, and IoT applications [21,39,40,41]. However, all of them rely on costly computational methods or require storing long sequences of data. Conversely, Silva et al. [23] proposed a lightweight unsupervised method to tune algorithms for time series. Nevertheless, this method is limited to drift detectors, requiring specific changes to trigger the tuning process.

Concerning recommendations, an example is the model-agnostic meta-learning proposed by [42], which learns an initial set of parameters for the model (in general, a neural network). In this way, it can solve new learning tasks and be applied to a range of different models, achieving relevant performance. Another work in this area is presented by [43]. It proposes an algorithm built on meta-learning to learn from the prior distribution of the model parameters. The authors stated that applying MtL for recommendation speeds up model adaptation and improves performance.

In comparison to the mentioned works, our meta-learning proposal provides a solution focused on CUSUM. Considering its particularities mapped by particular meta-features we propose a more robust and efficient approach by leveraging knowledge from related tasks, enabling faster adaptation, reduced computational costs, and improved model performance across diverse time-series challenges, including hyperparameter optimization [37,42,43], concept drift detection [1,35,36], and recommendation systems [44,45,46].

## 3. Problem Statement

Due to the dynamical world, approaches must handle nonstationary distributions, i.e., detecting concept drift. The definition of concept drift refers to statistical properties of the target changes over time in unforeseen ways [47]. According to [48,49], given a period of time, the concept drift can be formally represented between two time points (*t* and t+1) by:

(1) $$\exists X:P_{t}\left(X,y\right)\neq P_{t+1}\left(X,y\right)$$

where $P_{t}$ represents the joint distribution at time *t* between *X* (set of input variables) and *y* (target variable); in this sense, $P_{t+1}$ denotes the joint distribution at time t+1. Concept drift is related to covariance shift in the distribution, i.e., at time *t*, it can be inferred that concept drift refers to the joint probability change in *X* and *y*. Thus, $P_{t}\left(X,y\right)$ can be defined as a composition of $P_{t}\left(X\right)\times P_{t}\left(y|X\right)$. In this context, concept drift refers to changes in comparison to input data and output, resulting in a variation in the distribution. In this paper, we focused on abrupt concept drift and its impact on predictive performance. To provide reliable results, timely detection was considered essential.

### 3.1. The Cumulative Sum Algorithm

CUSUM [7] is a statistical technique based on the Sequential Probability Ratio Test (SPRT) [50]. This algorithm is the well-known change-point detector and assumes the distribution before and after the change-point occurrence is completely specified. Suppose that the sequential observations $x_{i},i=1,2,\ldots inf$ represent the observed signal. The upper CUSUM statistic is defined as g(t). Set g(1)=0. For a chosen $\gamma_{c}$ and *h*, drift and threshold, respectively, the time of a change in the signal r(t) is estimated by observing when $g\left(t+1\right)=max(g\left(t\right)+x_{i}-\gamma_{c},0)$ exceeds *h*. After a change has been detected, *g* is reset to zero, and the last *t* for which g(t)=0 is taken as an estimate of the change time. The hyperparameter $\gamma_{c}$ called drift is related to drift correction. Thus, it prevents false positives in the absence of an actual change or a slow drift.

The CUSUM algorithm detects the mean shift of the signal by checking when the test statistic *g* exceeds some threshold *h*. Summing consecutive signal values, a large *g* indicates the mean has changed. Therefore, CUSUM is a sequential analysis technique that accumulates deviations from target values. For that, there must be a correct hyperparameterization for each scenario.

Based on the literature, we inferred there are many improvements in terms of the MtL process and hyperparameters’ recommendation. Regarding time-series analysis and CUSUM, the change-point detection poses challenges not only about the signal structure but also that the MtL process cannot be costly. Therefore, the proposed approach implies identifying suitable hyperparameter values for the CUSUM in order to automate its process.

### 3.2. Meta-Learning for Tuning

Given a task from a plethora of options, the basic configuration and the appropriate steps to hyperparameters’ recommendation built on MtL can be designed. The pipeline is built on two macro steps: (1) Meta-learning modeling; and (2) Meta-learning predictions. The first step consists of learning from historical information. Regarding the second step, ML aims to automatically predict a suitable configuration for algorithms to predict them according to the input data.

Formally, let A denote the CUSUM algorithm associated with a hyperparameter space $H=\{H^{\left(1\right)},\ldots ,H^{\left(n\right)}\}$. A vector of hyperparameters for A is denoted by λ∈Λ, where A×H⊆Λ. Given a data set P composed of meta-features from a distribution D, we have l combinations of CUSUM and respective hyperparameters. As we are facing a multi-output problem, Λ is associated with a single instance.

The hyperparameters’ recommendation problem is described as how to find a function able to map M:P→Λ. There are several ways and alternatives for modeling the mapping function. In this paper, we followed through meta-learning, which is considered a fast solution and task-independent for not requiring information from the input data set at hand [51]. In summary, the pipeline consists of data preparation (metadata), inducting ML models to learn from historical information, and then tuning ML algorithms.

In the context of meta-learning, a meta-database is a repository that stores meta-examples, each representing a learning problem along with the performance metrics of various algorithms applied to it. Each meta-example comprises a set of descriptive features, known as meta-features, which characterize the problem’s properties, and a target attribute, referred to as the meta-target, indicating the optimal algorithm or performance outcome for that problem. This structure facilitates the analysis and selection of suitable algorithms for new problems by leveraging prior knowledge encapsulated in the meta-database [52].

## 4. Experimental Methodology

We evaluated the performance of the proposed approach by assessing the effectiveness of CUSUM detection using detection metrics, specifically the F1-Score. It is worth noting that our proposal offers an integrated solution for hyperparameter tuning; however, our primary focus is on improving the drift detection rate. Since we want to explore how chosen hyperparameters affect the performance and propose a meta-modeling, we use straightforward comparison using CUSUM to provide an overview of meta-recommendation achievements when considering both hyperparameters ($\gamma_{c}$ and *h*). Regarding the whole process, the pipeline is built on two macro steps: (1) meta-learning modeling; and (2) meta-learning predictions. The first step consists of learning from historical information. Regarding the second step, ML aims to automatically quickly find a better set of ML models by predicting them according to the input data. In summary, the pipeline consists of data preparation (metadata), inducting ML models to learn from historical information, and then tuning hyperparameters from ML algorithms.

**Figure 1:**
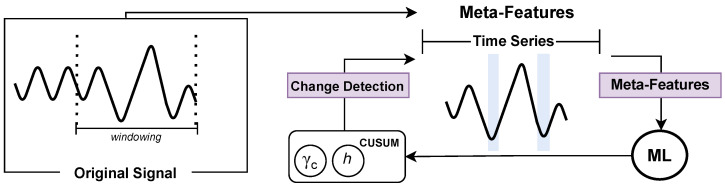
Overview of the problem and experimental framework.

In order to obtain a robust and well-performing pipeline, we propose the architecture shown in Figure 1. The framework is fashioned in five steps: (1) Metadata preparation; (2) Meta-feature extraction; (3) Meta-model induction; (4) Hyperparameters’ prediction; (5) Evaluation step, which defines the meta-targets, the classes/labels to be predicted by the MtL recommender system. In summary, it checks for abrupt change detection in the original signal.

In this context, we propose a framework, which is built on meta-models to configure CUSUM’s $\gamma_{c}$ and *h* hyperparameters to detect change points in time series. An MtL approach was introduced to generate metadata that supports the automatic selection of CUSUM hyperparameters, enabling faster adaptation than GS while maintaining accuracy. The next subsections present the proposed approach in detail, the datasets employed in this work, the evaluation metrics used, and the experimental setup.

To assess the impact of different meta-feature domains on the meta-learning process, we conducted an analysis of the extracted meta-features using the TSFEL (Time-Series Feature Extraction Library) package. This analysis aimed to evaluate how different types of meta-features contribute to both predictive performance and computational efficiency. The extracted meta-features were categorized into statistical, temporal, and spectral domains, and experiments were conducted selectively using different combinations of these feature sets.

In this study, we defined seven experimental setups to examine the effect of each meta-feature category: (1) Using all available TSFEL features; (2) Using only statistical meta-features; (3) Using only temporal meta-features; (4) Using only spectral meta-features; (5) Combining statistical and spectral features; (6) Combining statistical and temporal features; and (7) Combining temporal and spectral features. Each of these configurations was evaluated in terms of F1-Score and processing time.

### 4.1. Metadata Preparation and Meta-Feature Extraction

Data preparation is a typical process usually adopted in ML pipelines. Although inspired by human learning, our proposal involves MtL techniques to compose our dataset. Regarding the MtL workflow in this work, it is considered a learning process applied to metadata, which consists of a large set of features extracted from the input time series. This input signal consists of a time series from a fixed-size slide window. Each part of this distribution represents a sub-dataset of the previous one. Following a divide-and-conquer strategy, we segment the input time series into smaller, fixed-size windows. From each segment, relevant statistical, temporal, and spectral characteristics are extracted to compose the metadata, ensuring a detailed representation of the time-series dynamics.

Due to ML pipelines, features must be extracted from time series to turn them into a set of properties that characterizes the distribution. Composing the second step, those features were obtained from TSFEL. Introduced by [53], this package computes features across temporal, statistical, and spectral domains built to provide fast exploratory data analysis and feature extraction. Then, the extracted features, called meta-features, are combined with meta-targets, which are the most suitable CUSUM hyperparameters collected based on GS’s performance. For each window, we saved the set of hyperparameters that had the best performance for the respective time series. Using the meta-database, the MtL step induces a meta-model.

### 4.2. Regression Meta-Model (Recommender)

Following the framework, the third step is related to training the ML algorithm. Since the proposed method is based on MtL strategies, an associated base-learner is required. Regression models provide the final decision of a given sample based on its feature vector. Several of them were selected based on their proven capabilities, interpretability, and suitability in related problems and because there are well-known algorithms with relevant results in different problems, as follows: Decision Tree (DT), which is widely applied to represent a series of rules that lead to a class or value. It was chosen due to its interpretability and straightforward handling of nonlinear relationships, essential for meta-learning tasks involving clear rule-based decision making [54,55]; Random Forest (RF) was included for its robustness, as it aggregates multiple DTs, reducing variance and improving generalization capabilities. It is particularly advantageous in managing high-dimensional metadata and noisy datasets and provides more accurate predictions [56,57]; Support Vector Machine (SVM), which is a statistical learning algorithm, used for supervised ML, was selected due to its effectiveness in handling complex patterns and providing stable predictions in high-dimensional feature spaces. SVM excels in achieving precise regression outcomes [58,59].

By explicitly outlining these choices, our approach benefits from the complementary strengths of these models, enabling a robust and comprehensive meta-learning solution for hyperparameter recommendation in time-series analysis. This strategic combination aligns seamlessly with the framework’s overall objectives, supporting the adaptability required for handling diverse time-series scenarios and effectively addressing practical challenges such as computational efficiency, real-time responsiveness, and adaptability to concept drift. Moreover, employing different models allows us to explicitly validate the advantages and limitations of each, thereby providing insights into their performance boundaries and ensuring a more informed and balanced selection strategy in practical applications.

### 4.3. Datasets

To compare the classical CUSUM and the proposed solution, predictive performance, and time processing were considered. For that, we adopted benchmark datasets. The benchmark datasets include both real and synthetic ones that are used in the literature in general, which were collected from various online sources, including WorldBank, Eurostat, GapMinder, and Yahoo Finance. Before performing the experiments, all datasets underwent meticulous preprocessing to ensure the reliability and reproducibility of our findings. The preprocessing pipeline involved initial data cleaning, including the removal of missing values and anomalies identified through exploratory data analysis. Subsequently, normalization techniques were applied to standardize the scales across the different features, thus avoiding potential biases during meta-model training and ensuring consistent feature contributions. Table 1 summarizes the main aspects of each dataset used, outlining the number of samples, abrupt changes, and the percentage of detected change points.

### 4.4. Additional Optimization Methods

In addition to the proposed framework, two additional methods were explored in this experiment: Grid Search and Genetic Algorithm optimization. These methods were chosen because they are used in most of the hyperparameter optimization studies [61,62,63].

Regarding the Genetic Algorithm, it was conducted using an evolutionary algorithm software modeling package called Distributed Evolutionary Algorithms in Python 3.8 (DEAP) [64]. Selection was performed by roulette selection. Additional runtime parameters were determined empirically, which are: population size—10; generations—100; mutation rate—0.1; crossover rate—0.5.

For the Grid Search, at each step, all possible combinations were analyzed, storing the best performance, considering F1-Score and time processing. However, to choose the best combination, meta-models were used, as described in Section 4.2. To improve the exploratory analysis, not only a single meta-model for each hyperparameter was inducted, but also multi-output regression meta-models to predict both.

The main contributions made by this research, supported by the experimental results, were summarized under four perspectives. First, the results were presented considering the predictive performance of the meta-learners and the two traditional hyperparameters’ optimization previously mentioned (GS and GA). Second, further details were explored, and the impact of the recommendation procedure was discussed. Afterward, the meta-feature influence on the recommendation was assessed. Finally, an analysis was conducted considering the advantages and drawbacks of the proposed hyperparameters’ optimization framework.

Among the deterministic approaches, the GS is the most basic hyperparameters’ optimization method due to its simplicity. Also known as full factorial design, GS is fashioned on a finite set of values for each hyperparameter. To do so, it evaluates the Cartesian product of these sets to find a suitable one. However, GS is an exhaustive search method that requires the democratization of the hyperparameter space. It suffers from the number of function evaluations that grow exponentially with the dimensionality of the configuration space. Regardless of its drawbacks, it is still the most used method in the literature [65,66].

Considering probabilistic optimization methods, GA is a population-based method that evolves its population by applying mutations and crossover to generate a better configuration. In tuning tasks, it can be useful for complex optimization problems that require a large number of hyperparameters. Therefore, GA helps to find an optimal set of values in a domain [67].

### 4.5. Implementations

To carry out the experiments, the Python language was used, as well as the respective standard libraries for data manipulation. Taking into account the CUSUM algorithm, the detecta package was used, which is a module that provides the implementation of CUSUM. The CUSUM algorithm was used to identify the change points in the time series. One of the ways to implement CUSUM is to calculate the Cumulative Sum of positive and negative changes, comparing it with a threshold, where, if this threshold is reached, the change is detected, and the alert occurs, then the sum is reset, and the analysis starts again. In addition, CUSUM also has a hyperparameter called drift, which is introduced to avoid detecting changes in the absence of a real or slow change. Thus, the CUSUM’s performance in detecting change points is directly related to the input set of hyperparameters. As a default in the experiment, the values of h=[1,30] were used for threshold while for the drift, we used: $\gamma_{c}=\left[0.1,1\right]$. From these, the performance analysis was elaborated.

### 4.6. Evaluation Metrics

The performance was evaluated from different scenarios through the F1-Score, which is calculated through the Precision and Recall. Precision and Recall are calculated by analyzing the Confusion Matrix. The respective diagonal values of this matrix are True Positive (TP) and True Negative (TN), which are divided by the sum of the values of the entire matrix (*n*). Recall and Precision are often used to evaluate the effectiveness of classification methods based on False Negative (FN) and False Positive (FP). In this work, these metrics were used for a fair comparison of the quality of the results obtained. Furthermore, the processing time from metadata preparation to prediction was computed.

For a fair analysis, the metrics were compared to the GS and the GA optimization to find a suitable set of hyperparameters and optimize them, respectively. Thus, it is possible to estimate the overall work execution with an additional perspective of performance analysis. In the experiments, the time consumption was calculated as the average of 30 runs.

## 5. Results and Discussions

### 5.1. Predictive Performance

The predictive performance obtained by the proposed approach, the baseline methods, and each time consumption is presented in Table 2. Observing each performance, in general, we infer that the best results were obtained from GS and GA methods, as expected. Regarding the Grid Search, it happens because all the possibilities in the predefined scenario were combined and tested in a sort of grid. The combination that yielded the best performance was selected. Considering GA’s performance, as it is based on an evolutionary process, it is efficient in comparison to traditional methods. Due to its optimization capabilities, both continuous and discrete values are tested to find an optimal result, which is an advantage in comparison to GS. However, it is time-consuming.

From meta-models, some particular results can be observed. As a single meta-model for each hyperparameter, multi-output meta-models were explored. When time processing is considered, smooth variability can be observed. Due to the small number of hyperparameters, the discrepancy between these methods is low. However, as the set of hyperparameters increases, this difference tends to be more expressive.

Comparing GS and GA optimization to each meta-model, regardless of dataset or meta-model, the proposed framework is less time-consuming than those. This is mostly related to the fact that, after the induction process, as meta-models learn from a meta-dataset, to predict them is faster than finding out each set of hyperparameters to each time series, as the training process is executed only once.

### 5.2. Analysis of Recommendation Procedures

**Figure 2:**
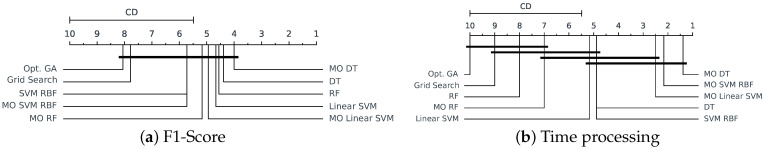
Comparison of F1-score and time performances among the algorithms, and the meta-model versions with multi-output according to Nemenyi test with α=0.05.

We also used the Friedman statistical test [68] to compare the performance of each strategy. Then, we used the Nemenyi post hoc test [69] with confidence 0.05 to create the critical distance (CD) diagrams in Figure 2a,b, for F1-Score and time processing.

The evaluation of predictive performance using the F1-Score suggests no statistically significant differences among the tested methods (GS, GA optimization, and the proposed meta-learning models) at α=0.05. This indicates that the proposed HPO framework achieves comparable accuracy to GS and GA, both of which are widely regarded as strong optimization baselines. However, a different trend is observed when analyzing computational efficiency. Statistically significant differences were found in time processing across all methods, highlighting the advantage of the proposed approach. Among the tested strategies, multi-output meta-models demonstrated the most conservative behavior in terms of computational cost, followed by single meta-models trained for each hyperparameter. Notably, the rank differences in processing time are substantial, reinforcing the impact of computational efficiency in hyperparameter selection.

When examining the practical implications of these findings, it becomes evident that the proposed HPO framework not only maintains high predictive accuracy comparable to GS and GA but also significantly reduces processing time due to its meta-learning-based design. This reduction in computational overhead is a crucial advantage, particularly in dynamic environments where rapid hyperparameter tuning is essential for real-time applications.

### 5.3. Meta-Feature Analysis

**Figure 3:**
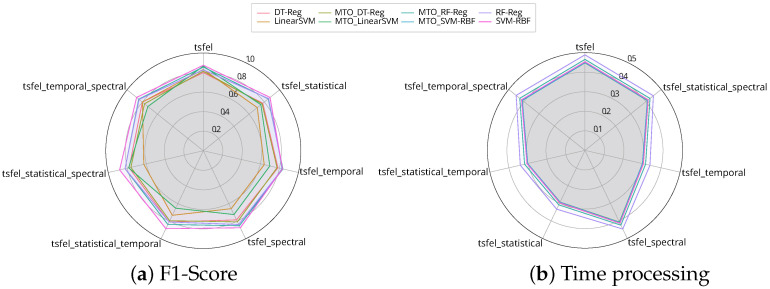
F1-Score and time processing from each meta-feature from TSFEL library.

Taking advantage of the MtL framework for further analysis, we observed the impact of each meta-feature in inducting meta-models. Figure 3 shows seven different cases regarding each meta-feature domain and each evaluated metric: (1) TSFEL refers to extracting all domains from time series; (2) Extracting only statistical meta-features; (3) Only temporal ones; (4) Extracting meta-features in spectral domain; (5, 6, and 7) Combining each of the meta-features from TSFEL library.

Considering the predictive performance, we observed that the meta-features, regardless of the domain, did not significantly influence the performance. It is most related to the type of the meta-model. Instead, the meta-features domain had a significant effect on the time processing. As shown in Figure 3b, extracting all meta-features available in the TSFEL library and the spectral ones were the highest time-consuming due to their properties. The temporal was the lower focus on time.

The obtained results suggest that the meta-feature domains have considerable differences among them, and some are time-consuming. This study also shows that the predictive performance is similar for each case because these meta-feature domains did not influence the meta-model induction, indicating that the hyperparameters’ optimization framework might deal with different scenarios.

In our framework, we employed the TSFEL to generate comprehensive meta-features grouped into three primary domains: statistical, temporal, and spectral. Statistical features such as mean, variance, skewness, and kurtosis were particularly valuable in capturing the distributional properties of the data and contributed substantially to model performance by providing baseline characteristics of the time series. Temporal features, including autocorrelation and zero-crossing rate, captured the time-dependent behaviors and periodicities in the data, further enhancing the predictive accuracy of the framework.

Spectral features, such as spectral entropy and frequency-domain statistics, were instrumental in identifying patterns in the frequency domain, allowing the meta-model to detect subtle and complex variations in the time series. Our experiments indicated that combining statistical and temporal features generally resulted in higher predictive performance, suggesting their complementary nature in capturing different aspects of the time-series dynamics. Conversely, while spectral features added valuable insights, they significantly increased computational overhead due to the higher complexity of frequency-domain calculations.

Overall, this detailed meta-feature analysis highlighted that a carefully balanced combination of statistical and temporal features could effectively optimize both prediction accuracy and computational efficiency. Further research could focus on identifying feature-selection strategies to minimize computational demands while maintaining high predictive capability, particularly relevant for real-time applications.

### 5.4. MtL Framework: Advantages and Drawbacks

The results showed that there is a significant reduction in the time consumption when using the MtL framework. In particular, the best predictive results were achieved with GS and GA optimization. However, the proposed approach does not have statistical differences with these methods, which shows a considerable advantage and promising applications.

According to the reported results and focusing on time processing, the MtL framework demonstrated a superior capability to deal with different scenarios, maintaining the average time consumption. However, to achieve good performance, it requires an ideal set of hyperparameters in the metadata preparation stage, which might be considered a disadvantage. Given its reliance on meta-models, representative metadata, and target variables must be carefully prepared to ensure effective learning.

The primary advantage of the MtL framework lies in its structural simplicity. Unlike GA optimization, it does not require defining a complex objective function, simplifying implementation considerably. Furthermore, the MtL approach can leverage synthetic datasets with clearly identified change points to effectively train meta-models.

Despite these advantages, deploying the MtL framework in real-world streaming environments presents several challenges. One critical challenge is effectively managing concept drift in live-streaming data, where model accuracy may degrade over time if drift is not promptly and adequately addressed. Thus, ensuring the framework can continuously adapt through incremental updates without significant performance degradation is crucial. Additionally, addressing the computational resource constraints and latency considerations inherent in live data processing environments will be essential to ensure practicality and scalability in real-world scenarios.

Moreover, potential limitations exist regarding the generalizability of the MtL framework to unseen data distributions. Since the meta-learning approach heavily depends on previously observed metadata, it may encounter difficulties in accurately predicting optimal hyperparameters for datasets exhibiting characteristics significantly different from the training distributions. Thus, ensuring diversity and representativeness in the training metadata is vital to enhance the robustness and broader applicability of the framework.

## 6. Conclusions and Future Work

In this paper, we proposed an MtL framework to recommend a set of hyperparameters for CUSUM. For that, we have extracted meta-features to describe a time series and an ideal hyperparameter set was obtained based on GS to compose the meta-dataset. Concerning the highlighted subjects, the framework had no statistical difference in comparison to GS and GA optimization, which means that it has competitive predictive performance. Moreover, it has shown less time consumption than the others.

In future research, we aim to extend the experimental evaluation to other drift scenarios and types of concept drift to gather further insights into the proposed framework. Furthermore, we intend to investigate the integration of deep learning techniques into our MtL framework. Leveraging deep neural networks could enhance feature extraction and automated hyperparameter tuning processes, potentially improving prediction accuracy and further reducing computational overhead. Such an integration might enable the framework to dynamically adapt to complex and evolving time series data, making it more robust and effective for real-world applications.

Another promising direction involves extending our proposed MtL framework to other drift detection algorithms beyond CUSUM to evaluate its versatility and effectiveness across diverse methodologies. Exploring these extensions would validate the framework’s broader applicability and potentially contribute to a generalized solution for hyperparameter optimization in various concept drift detection contexts.

Additionally, future research should explore real-world deployment challenges, particularly addressing the handling of concept drift in live-streaming data environments. Practical deployments must consider the computational constraints and latency requirements inherent to real-time processing. The framework may benefit from incremental learning methods, capable of continuously updating the model without extensive retraining phases. It is also crucial to develop robust drift detection and adaptation mechanisms to maintain reliable performance over extended operational periods, thus enhancing the usability and applicability of the MtL framework in diverse industrial settings.

## Figures and Tables

**Table 1:** Summary of the datasets used in the experiment.

Dataset	# Samples	# Abrupt Changes	% Change Point
Bank [24]	581	0	0.00
Children per Woman [24]	301	7	2.33
CO2 Canada [24]	215	12	5.86
Quality Control 4 [24]	500	6	1.20
Quality Control 5 [24]	325	0	0.00
Ratner Stock [24]	600	7	1.17
Scanline 42049 [24,60]	481	21	4.37
USD isk [24]	247	9	3.64

**Table 2:** Mean values F1-Score and time for each combination of algorithm and dataset. We took advantage of the original and multi-output (MO) version of each algorithm. The bold values correspond to the best mean result for each metric in the dataset.

Dataset	MO	Algorithm	F1-Score	Time (s)
bank	-	Grid Search	**1.000**	0.615
		Opt. GA	**1.000**	0.915
		Linear SVM	**1.000**	0.310
		SVM RBF	**1.000**	**0.307**
		DT	**1.000**	**0.307**
		RF	**1.000**	0.342
	✓	Linear SVM	**1.000**	0.305
		SVM RBF	**1.000**	0.304
		DT	**1.000**	**0.303**
		RF	**1.000**	0.320
children_per_woman	-	Grid Search	**1.000**	0.590
		Opt. GA	**1.000**	0.935
		Linear SVM	**1.000**	**0.268**
		SVM RBF	**1.000**	**0.268**
		DT	**1.000**	**0.268**
		RF	**1.000**	0.297
	✓	Linear SVM	**1.000**	**0.264**
		SVM RBF	**1.000**	**0.264**
		DT	**1.000**	**0.264**
		RF	**1.000**	0.278
co2_canada	-	Grid Search	**0.846**	0.592
		Opt. GA	**0.846**	0.800
		Linear SVM	**0.846**	**0.272**
		SVM RBF	0.769	0.273
		DT	0.692	0.273
		RF	0.692	0.302
	✓	Linear SVM	**0.846**	0.269
		SVM RBF	0.769	0.269
		DT	0.538	**0.268**
		RF	0.769	0.283
quality_control_4	-	Grid Search	0.989	0.676
		Opt. GA	**1.000**	0.922
		Linear SVM	0.967	0.329
		SVM RBF	0.833	0.328
		DT	0.600	**0.324**
		RF	0.692	0.377
	✓	Linear SVM	0.500	**0.320**
		SVM RBF	**0.833**	0.324
		DT	0.538	0.323
		RF	0.633	0.337
quality_control_5	-	Grid Search	**1.000**	0.596
		Opt. GA	**1.000**	0.930
		Linear SVM	0.450	**0.259**
		SVM RBF	0.750	**0.259**
		DT	0.650	**0.259**
		RF	0.650	0.288
	✓	Linear SVM	**0.850**	0.255
		SVM RBF	0.750	0.255
		DT	0.750	**0.254**
		RF	**0.850**	0.269
ratner_stock	-	Grid Search	**1.000**	0.648
		Opt. GA	**1.000**	0.920
		Linear SVM	0.500	**0.324**
		SVM RBF	**1.000**	**0.324**
		DT	0.700	0.327
		RF	0.767	0.363
	✓	Linear SVM	0.500	0.321
		SVM RBF	**1.000**	**0.320**
		DT	0.733	**0.320**
		RF	0.833	0.337
scanline_42049	-	Grid Search	0.822	0.646
		Opt. GA	**0.856**	0.832
		Linear SVM	0.689	**0.304**
		SVM RBF	0.689	**0.304**
		DT	0.789	**0.304**
		RF	0.692	0.339
	✓	Linear SVM	**0.822**	0.302
		SVM RBF	0.689	**0.300**
		DT	0.656	**0.300**
		RF	0.689	0.317
usd_isk	-	Grid Search	**1.000**	0.614
		Opt. GA	**1.000**	0.918
		Linear SVM	**1.000**	**0.287**
		SVM RBF	**1.000**	**0.287**
		DT	**1.000**	**0.287**
		RF	**1.000**	0.321
	✓	Linear SVM	**1.000**	0.283
		SVM RBF	**1.000**	0.283
		DT	**1.000**	**0.282**
		RF	**1.000**	0.299

## Data Availability

Data are contained within the article.
